# Relationship between Muscle Strength and Gait Parameters in Healthy Older Women and Men

**DOI:** 10.3390/ijerph20075362

**Published:** 2023-04-01

**Authors:** Andreas Stotz, Daniel Hamacher, Astrid Zech

**Affiliations:** 1 Department of Human Movement Science and Exercise Physiology, Institute of Sport Science, Friedrich Schiller University Jena, Seidelstraße 20, 07749 Jena, Germany; astrid.zech@uni-jena.de; 2 Methods and Statistics in Sports, Institute of Sport Science, Friedrich Schiller University Jena, Seidelstraße 20, 07749 Jena, Germany; daniel.hamacher@uni-jena.de

**Keywords:** relative strength, gait speed, stride time, stance time, stride length and step width

## Abstract

Maintaining sufficient muscle strength is fundamental to prevent a decline in basic physical functions such as gait, and is therefore a prerequisite for a healthy independent life in older people. However, the relationship between gait parameters and the strength of single muscle groups is reported with inconclusive results. The objective of this study was to analyze the relationship of strength of nine single muscle groups of lower and upper leg muscles as well as handgrip strength for gait parameters in older adults. Sixty-nine independently living older adults participated in the study. Maximum ankle plantar- and dorsiflexion, knee flexion and extension, as well as hip abduction, adduction, flexion, and extension strength, were measured using an isokinetic dynamometer. Additionally, hand grip strength measured via a hand dynamometer was obtained. Walking gait parameters were recorded with a 3D motion capture system on an instrumented treadmill. The relationships between multiple strength and gait variables were analyzed by Pearson’s correlation coefficient. Linear regression analyses were performed to identify the predictive ability of muscle strength (normalized to body weight) for gait speed, stride time, stance time, stride length and step width. Multiple significant weak to moderate positive ([r = 0.343, *p* = 0.047]–[r = 0.538, *p* = 0.002]) and negative ([r = −0.340, *p* = 0.046]–[r = 0.593, *p* = 0.001]) correlations that were unequally distributed between both sexes were detected. Significant regression models explained ([r^2^ = 16.6%, *p* = 0.015]–[r^2^ = 44.3 %, *p* = 0.003]) and ([r^2^ = 21.8%, *p* = 0.022]–[r^2^ = 36.1%, *p* = 0.044]) of the gait parameter variations for men and women, respectively. The results suggest a sex-specific relevance of single muscle groups for all gait parameters. This may be attributed to anatomical differences and it is important to prevent strength-related changes in gait parameters.

## 1. Introduction

Stable gait is an essential requirement for various activities of daily life and also for an independent lifestyle. With advanced age, gait parameters start to change. Step and stride lengths become shorter, resulting in slower preferred and maximum walking speed [1,2,3]. Older adults show reduced cadence, longer double support time, and prefer to walk with a 41 % wider step width than their younger counterparts [4,5]. These changes interact with an age-related decline in general physical function. Both strength [6,7] as well as gait impairments [8] are related to an increased risk of falling among older people and are, therefore, the focus of preventive exercise strategies. Gait speed is the most common gait parameter in geriatric research, but spatiotemporal parameters also help to identify other aspects related to gait function decline [5].

Muscle strength loss is one of the established influencing factors for diminished gait performance in older adults above the age of 60 [9,10,11]. Decrement in muscle size, progressive loss of type IIb muscle fibers, changes in pennation angles, slower muscle metabolism, inadequate neuromuscular activation, and the manifestation of intermuscular adipose tissue are factors potentially responsible for age-related strength loss [12].

Multiple muscle groups are involved during the complex process of walking. Most studies that investigate the relationship between muscle strength and gait parameters often limit their assessment to hand grip or knee extension strength [11,13,14]. The studies that measured multiple lower body muscle groups reported inconclusive results regarding their relationship with gait parameters [15,16,17,18,19,20,21].

One possible explanation is the heterogeneity of the measured study sample. Although a considerable number of studies reported differences in gait parameters between female and male older adults [9,16,22,23,24], nothing is currently known about whether the muscle strength and gait relationship is also sex-specific. Only recently, the possibility has been indicated that both sexes also differ regarding the relevance of specific muscle groups for gait parameters [16]. For instance, gait speed regulations are performed differently between sexes, with men using longer step lengths and women using faster cadence [25]. In addition, kinematic differences, e.g., greater pelvis excursion and rotation [26,27,28], as well as a greater ankle range of motion in the sagittal plane in women compared to men, may influence sex-specific differences in muscular demands during walking [26]. Therefore, the lack of distinction between both genders in data analysis [17,19,20] may have masked the effects for male and female participants and could have led to conflicting results on the relationship between muscle strength and gait parameters in the other studies.

To address the aforementioned research gaps, this study aimed to comprehensively examine the relationship between the strength of multiple lower body muscles with gait parameters in older male and female adults during normal gait patterns. New insights into how muscle strength influences various aspects of human gait could improve gait intervention programs and anti-fall prevention.

## 2. Materials and Methods

### 2.1. Participants

Senior citizens were recruited through local newspaper advertisements. For inclusion, participants needed to be at least 65 years old and able to walk without assistance. Exclusion criteria were the presence of neurological diseases such as Alzheimer’s, stroke, or Parkinson’s disease. Furthermore, participants with severe orthopedic conditions such as severe foot deformities or leg length differences that might unnaturally influence gait or balance were excluded. Participants with artificial joints, prothesis or metal in their lower limbs were also excluded. Participants also had to be injury-free in their lower limbs for at least six months and show no higher risk for cardiovascular complications before the examination. Inclusion and exclusion criteria were surveyed in a telephone interview. Participants did not have to fast and were asked to maintain their habitual level of hydration. If urinary urgency was present, they had the opportunity to empty their bladder prior to the testing session. All testing sessions took place between 8 a.m. and 6 p.m.

Participants signed written informed consent, and ethical approval was obtained by the local Ethical Commission (protocol number: FSV 18/49).

### 2.2. Testing Procedures

A cross-sectional study design was used. Each participant attended a single test session in the biomechanics lab of the department. All tests were performed by the same investigator with experience in strength and gait assessment.

#### 2.2.1. Anthropometry

Height was measured with a stadiometer. Body weight and body composition (skeletal muscle mass, total fat mass, and body fat percentage) were obtained with the Inbody 720 (JP Global Markets GmbH, Eschborn, Germany). The bioimpedance analysis with the Inbody 720 has previously been validated as a tool for the assessments of total body and segmental body composition in adults [29,30].

#### 2.2.2. Gait Assessment

The subject’s preferred walking speed was first measured. Participants were asked to walk six meters, of which the central four were timed with a stopwatch, as they habitually do in their daily life. Gait kinematics were measured with a 3D motion capture system (Qualisys, Göteborg, Sweden). The laboratory setup included ten infrared cameras and two high-resolution video cameras. Twenty-six passive reflective markers were attached to specific positions (anterior superior iliac spine, posterior superior iliac spine, lateral side of the greater trochanter, femur lateral and medial epicondyle, proximal tip of the head of the fibula, most anterior border of the tibial tuberosity, lateral prominence of the lateral malleolus, medial prominence of the medial malleolus, distal aspect of Achilles tendon insertion on the calcaneus and dorsal margin of the first, second and fifth metatarsal head) of the lower extremities according to the work of Leardini et al. [31]. The static position of the markers was determined as the subjects stood in a neutral position. Next, participants were familiarized with the instrumented treadmill (B-CTM4-B07, Bertec Corporation, Columbus, OH, USA). At first, a slow walking speed of 1.0 m/s with a slow acceleration of 0.5 m/s^2^ was set in the treadmill Bertec software and participants were instructed to hold on to the railing. With this setup, participants could get used to the moving ground and balance issues were mitigated. When the participants became more comfortable and were able to walk without the assistance of the railing, the walking speed was incrementally increased until the preferred overground walking speed was reached. After a stable gait pattern was visible, the participants kept walking for another minute, and then the two minute test trial was started.

#### 2.2.3. Strength Assessment

The isokinetic concentric muscle strength of the dominant leg was measured in the following order with Isomed 2000 (Isomed 2000 ^®^, D&R Ferstl GmbH, Hemau, Germany): (1) ankle plantarflexion and dorsiflexion (tested joint range −20° of dorsiflexion to 35° of plantar flexion), (2) knee extension and knee flexion (tested joint range 5° to 90° of knee flexion), (3) hip abduction and hip adduction (tested joint range 0° to 60° of hip abduction), (4) hip extension and hip flexion (tested joint range 10° to 100° of hip flexion). Leg dominance was determined by the personal preference to kick a ball. The assessments were performed at an angular velocity of 60° per second. The investigator adjusted the device to the anthropometrical dimensions of the subject for each muscle group. Afterwards, the pivot point of the joint and of the device was arranged accordingly. Before testing, each participant was fixed in the correct testing positions with straps to eliminate extraneous and compensatory movements. After the preparatory measures were completed, the full range of motion of the tested joint was examined and adjusted when needed. Before starting the test trials, participants underwent a familiarization phase, where they could perform the movements with increasing but submaximal effort. In addition, the subjects became familiar with the graphical feedback curves of the dynamometer, which were important to be able to smoothly transition between flexion and extension. Before testing, the subjects rested for 90 s. The testing phase consisted of two trials with three maximal repetitions per trial with 2 min of break in between. A third trial was performed in cases of a high torque difference between trials. Three repetitions were performed in each direction (flexion, extension, adduction and abduction) so that the participant had a sufficient number of attempts to display maximum strength. The maximum value from the two trials was used for data analysis. Participants were verbally encouraged by the tester during the trials. The results were presented as newton meters and were divided by body weight for further analysis.

Additionally, as commonly practiced in geriatric research [11,13,14], handgrip strength was measured with a hand dynamometer, i.e., the Seahan SH5001 (SAEHAN Corporation, Masan, Korea). Maximum hand grip strength over two trials was recorded to the nearest 0.5 kg in an upright standing position with the elbow flexed at 90 degrees. All participants used the hand dynamometer adjusted at the second handle position for grip strength assessment according to Trampisch et al. [32].

### 2.3. Data Processing

Data acquisition was performed with Qualisys Track Manager (version 4.3.0.0 Qualisys, Göteborg, Sweden). The fill level of each marker was maximized via manual marker assignment or automatic gap filling. A minimum marker detection level of 90% was achieved for all participants. With a sampling rate of 200 Hz and a measurement duration of 120 s, each data set consisted of 24,000 data points for each marker. Data processing and export were conducted with Visual 3D software (C-Motion Inc. Germantown, MD, USA). Further processing was conducted with MATLAB software (version R2014a, MathWorks, Natick, MA, USA). The measured gait kinematics included stride time, stance time, stride length, and step width. Stride time was the duration between the initial contacts of two consecutive touchdowns of the same foot. Stance time was the time between the initial touchdown and the toe-off of the same foot. Consequently, the stride length was defined as the distance between two consecutive touchdowns of the same foot. Step width is the length from the midline midpoint of the current footprint to the midline midpoint of the previous footprint on the opposite foot.

### 2.4. Statistical Analysis

Mean and standard deviations are reported in the descriptive data. Data sets were checked for normality. Gender differences were analyzed with a Student’s *t*-test. In case the equal variance was violated, the Mann–Whitney U test was applied. Pearson’s correlation was applied to examine the relationship between the strength of each muscle group and each gait parameter. Correlations coefficients of 0.10 ≤ r ≤ 0.39 indicated a weak, 0.40 ≤ r ≤ 0.69 a moderate, and r ≥ 0.70 a strong correlation, as classified by Schober et al. [33]. A value of *p* < 0.05 was taken as significant. Furthermore, linear regression analysis used to examine the predictive ability of significant strength measures for gait parameters was performed.

All statistical analyses were performed with JASP (version 0.14.1, JASP Team 2020, Amsterdam, The Netherlands) and R (version 4.1.3, R Core Team, 2020, Vienna, Austria).

## 3. Results

Sixty-nine older adults (35 men, 34 women) met the study inclusion criteria and participated. Table 1 shows the demographics, anthropometry and strength data for the male and female participants. Men were significantly taller (*p* < 0.001), heavier (*p* < 0.001) and had more skeletal muscle mass (*p* < 0.001) than women, but had less fat mass (*p* = 0.032) and a lower body fat percentage (*p* < 0.001). For all the measured muscle groups of the lower body, men showed significantly higher relative strength than women (*p* < 0.010). Table 2 presents spatiotemporal gait parameters. Men showed a significantly longer stride length (*p* < 0.001) than women. The Pearson’s correlation coefficient between all lower extremity muscle groups and gait parameter means (Table 3) revealed multiple significant weak to moderate positive (r = 0.343–0.538) and negative (r = −0.340–0.593) correlations that were unequally distributed between men and women.

The linear regression analysis (Table 4), performed for all parameters that reached significance in the correlation analyses demonstrates different the predictive nature of strength variables for gait between men and women. For men, all the models reached the level of significance and depending on the number and predictive ability of the independent variables (strength values), a range of r^2^ = 16.6–44.3% of the variation in the gait parameters could be explained. For women, no regression between the strength parameters and stride time was performed, since no significant correlations were found. Two of the four regression models reached the level of significance, explaining r^2^ = 21.8–36.1% of the gait parameter variation in women.

## 4. Discussion

This study examined the relevance of different muscle groups on spatiotemporal gait parameters in older adults during normal walking. The results show that the maximum strength of multiple muscle groups correlated with most gait parameters at a weak to moderate level, supporting the findings of other studies regarding the relationship between lower body strength and gait parameters [15,16,17,18,19,20,21]. Our study also revealed sex-specific aspects of the relationship of the different muscle groups. In previous reports, either only one sex was measured, or the results of both sexes were not analyzed separately [15,19,21].

From a functional standpoint, one could expect that the muscles operating in the sagittal plane (hip flexors/extensors, knee flexors/extensors; plantar/dorsiflexors) are responsible for propulsive forces and influence gait speed [15]. This assumption is confirmed for men where gait speed was predicted by plantar flexors, knee extensors, and hip extensors. However, in women, not only these muscle groups but all other muscle groups, except dorsiflexors, were also predictors for gait speed. One possible explanation might be the higher muscle strength demands for women in order to accomplish a similar walking speed to men who have significantly higher strength in all muscle groups. This may be explained by the previously described increases in muscle co-activation during gait in elderly adults, which are compensatory mechanisms due to an increase in walking energy cost [34] or to increase joint stiffness that thereby enhances stability [35]. However, it is still unknown if and how sex-specific aspects can influence those mechanisms. Gait speed is generally determined by stride length and stride time [36]. Both gait parameters are differently related to lower body muscle strength between men and women. None of the measured muscle groups were identified as predictive factors for stride length in men. In women, however, a higher hip and knee extension strength predicted greater stride length. Contrary results were found for stride time predictability. Shorter stride times in men were related to the strength of nearly all knee and hip muscles, while there was no relationship between muscle strength and stride time in women. It seems that lower body muscle strength is less relevant for stride length but more for stride time at normal walking speed in men, while this relationship seems to be reversed for older women. Men generally exhibit a longer stride length but a slower cadence compared to women [25]. In a recent study, it was reported that some older adults increase their step length and others decrease their step time to walk fast, but it is not clear whether those strategies are preferably adopted by one sex or the other [36]. Therefore, we suggest that further research is necessary to examine the sex-specific stride length and stride time interaction and whether this can be influenced by improvements in lower body strength from regular exercising.

Muscles that primarily function in the frontal plane (hip abductors/adductors) are supposed to be related to step width [37]. This is supported by our finding, since the hip abductors predicted step width in both the female and male participants. However, in women, multiple other ankle, knee and hip muscle groups were significantly related to step width, while none of these additional muscle groups predicted step width in men. Again, this could probably be explained as a compensatory mechanism for possible strength deficits of single muscle groups in women [38,39] when a sufficient walking speed is maintained. Additionally to the lower limb muscles, we included a grip strength measurement as is commonly used in geriatric research [11,13,14]. Although it is an unspecific strength test, hand grip strength was related to gait speed and step width in women and was the only strength parameter related to stride length in men. It seems that hand grip strength also has a sex-specific relationship to gait parameters, but further research is necessary to examine why only certain gait parameters are related to hand grip strength.

The sex-specific differences in muscle strength and gait relationship are probably due to different reasons. It has been recently proposed that normalization of gait parameters to body size or leg length can diminish sex-specific differences [25,27]. In their meta-analysis, Frimenko et al. [25] concluded that gait speed differences between men and women may be an artifact of size rather than sex. According to their data, men and women, even after accounting for differences in size, control gait speed differently, with men using longer step lengths and women using faster cadence. However, the authors also observed a change among metrics throughout an adult’s lifespan, emphasizing the need for further analysis among different age groups. In a study with healthy young adults [26], the sex differences in spatiotemporal metrics and center of mass displacement disappeared when we controlled for size, while the ankle, pelvis and torso range of motion persisted or even increased with faster gait speed.

Different anatomical and physiological parameters might also influence sex-specific differences in muscular demands during walking. In women, the pelvis is shorter, broader and in a more anterior position and executes greater excursion and rotation movement compared to men, who have a more neutral position of the pelvis [26,27,28]. It has been suggested that these greater ranges of motion and pelvis dimensions lead to longer strides relative to their leg length [40]. This strategy might explain the sex-specific relationship of knee and hip extensor muscles with stride length in our results. Ankle kinematics also tend to be different between men and women, with a greater range of motion in the sagittal plane in women compared to men across multiple walking speeds [26]. This may related to the higher gastrocnemius lateralis activity with a woman’s gait when compared to men [41]. It is also assumed [27] that the proportionally shorter female foot length [42,43] requires a greater plantarflexion push-off angle in order to reach the same vertical height change as a longer foot [26]. The current and prior findings highlight the importance of sex considerations for gait analysis studies [24,27].

The present study contributes to the understanding of how muscle strength is related to gait control in healthy older adults and may help to manage strength-related changes in gait parameters. Some strength intervention studies that targeted the lower body have reported increases in gait speed in older adults, but not examined how the gait parameters have changed in the process [44]. Further research should examine (1) which gait parameter changes are associated with improved gait abilities after resistance training interventions, (2) if adaptations are sex-specific and (3) if strength improvement of a certain muscle group is related to a specific gait pattern, as shown in our results.

### Limitations

While this study presents novel insights, a few limitations need to be addressed. The methods section does not provide a sample size calculation; however, due to the magnitude of the measured and correlated parameters, a sample size calculation should be performed to match the estimated and measured sample size. We analyzed gait parameters while walking on an instrumented treadmill, using more than 100 gait cycles over two minutes. However, this approach limited us to only examining preferred paced walking. It was uncertain how long subjects could sustain faster or the maximum walking speed safely on the instrumented treadmill. Nevertheless, muscle strength seems to be more closely related to faster gait speed than normal walking speed [19]. Therefore, it seems plausible that for fast walking, the predictive ability of muscle strength for gait parameters might also increase [14]. This aspect is important for senior citizens to maintain independence in situations of daily living when faster walking speeds, e.g., crossing a street, is required [45]. In addition, walking on a treadmill differs from ground walking and changes the gait parameters [46,47]. This effect might be stronger depending on previous treadmill experiences. This lessens the transferability of the results on natural walking. Only concentric strength with one single angular velocity was measured; further research is necessary to investigate whether other contraction types or other contraction speeds can account for of the greater variance in the dependent variables. Lastly, strength assessments of nine separately measured muscle group with an isokinetic device present a certain amount of equipment bias and do not exactly represent muscular demands during walking. In addition, this approach provides no information regarding the cross-coordination (coupling) of several muscle groups during the complex task of walking.

## 5. Conclusions

The strength of multiple muscle groups in the lower body is related to multiple gait parameters. The results are sex-specific with no muscle groups when predicting the stride length in men and no muscle groups when predicting the stride time in women. The results may be attributed to sex-specific differences in anatomy, kinematics, and size, and it is suggested that research that examines the relationship between strength and gait parameters needs to consider sex-specific interactions. The results are of major importance to prevent strength-related changes in gait parameters in older adults.

## Figures and Tables

**Table 1:** Anthropometrics, relative strength, and gait parameters of male and female subjects.

	Total	*n* = 69	Men	*n* = 35	Women	*n* = 34	
	Mean	SD	Mean	SD	Mean	SD	*p*
**Characteristics**							
Age (y)	76.67	4.72	76.80	4.66	76.53	4.77	0.814
Height (m)	166.80	8.93	172.66	6.52	160.77	6.73	**<0.001**
**Body Composition**							
Weight (kg)	72.42	11.54	77.29	9.29	67.41	11.46	**<0.001**
BMI (kg/m^2^)	26.08	3.43	25.90	2.60	26.25	4.15	0.675
Skeletal muscle mass (kg)	26.42	5.25	30.54	3.55	22.17	2.67	**<0.001**
Total fat (kg)	23.79	7.89	21.79	6.44	25.84	8.78	**0.032**
Body fat percentage (%)	32.54	8.05	27.72	6.31	37.51	6.52	**<0.001**
**Muscle Strength**							
Grip strength (kg/kg)	0.43	0.10	0.48	0.10	0.37	0.07	**<0.001**
Ankle plantarflexion (Nm/kg)	0.75	0.23	0.81	0.24	0.69	0.21	**0.022**
Ankle dorsiflexion (Nm/kg)	0.16	0.06	0.18	0.05	0.13	0.05	**<0.001**
Knee flexion (Nm/kg)	1.05	0.26	1.22	0.19	0.89	0.22	**<0.001**
Knee extension (Nm/kg)	1.44	0.38	1.61	0.38	1.27	0.31	**<0.001**
Hip abduction (Nm/kg)	0.69	0.28	0.83	0.24	0.55	0.24	**<0.001**
Hip adduction (Nm/kg)	1.75	0.49	1.96	0.45	1.54	0.44	**<0.001**
Hip flexion (Nm/kg)	1.03	0.33	1.24	0.29	0.82	0.22	**<0.001**
Hip extension (Nm/kg)	2.73	0.73	3.16	0.65	2.29	0.49	**<0.001**

Significant correlations (*p* < 0.05, *p* < 0.01) are marked in bold. (Nm/kg) describes the muscle torque in newton meters relative to the participant’s body weight in kilograms.

**Table 2:** Spatiotemporal gait parameters for men and women.

Gait Parameters	Total	*n* = 69	Men	*n* = 35	Women	*n* = 34	
	Mean	SD	Mean	SD	Mean	SD	*p*
Prefered gait speed (m/s)	1.28	0.20	1.28	0.23	1.27	0.18	0.907
Number of strides (N/min)	116.51	11.64	115.31	10.08	117.74	13.10	0.392
Stride time (s)	1.01	0.09	1.02	0.09	0.99	0.10	0.223
Stance time (s)	0.60	0.07	0.61	0.06	0.59	0.07	0.485
Stride length (m)	1.06	0.20	1.13	0.18	0.98	0.18	**<0.001**
Step width (m)	0.10	0.03	0.11	0.03	0.10	0.03	0.113

Significant correlations (*p* < 0.05, *p* < 0.01) are marked in bold.

**Table 3:** Correlations (Pearson’s r) between the strength of lower limbs and gait parameters for men and women.

Muscle Force Parameters (Men)										
Gait Parameters		Plantarflex	Dorsiflex	Kneeflex	Kneeex	Hipflex	Hipext	Hipabd	Hipadd	Handgrip
Gait speed	Pearsons r	**0.365**	0.287	0.266	**0.415**	0.322	**0.492**	0.033	0.268	0.324
	*p*-value	0.037	0.106	0.134	0.016	0.068	0.004	0.857	0.132	0.066
Stride time	Pearsons r	−0.304	−0.187	**−0.347**	**−0.593**	**−0.422**	**−0.544**	−0.199	**−0.340**	−0.207
	*p*-value	0.076	0.283	0.041	0.001	0.012	0.001	0.251	0.046	0.232
Stance time	Pearsons r	**−0.388**	−0.214	−0.328	**−0.540**	**−0.437**	**−0.564**	−0.197	−0.255	−0.267
	*p*-value	0.021	0.216	0.055	0.001	0.009	0.001	0.258	0.139	0.122
Stride length	Pearsons r	0.311	0.316	0.093	0.2	0.285	0.302	0.006	0.18	**0.442**
	*p*-value	0.069	0.064	0.597	0.249	0.097	0.078	0.974	0.301	0.008
Step width	Pearsons r	−0.226	−0.007	−0.188	−0.002	−0.202	−0.134	**−0.407**	−0.118	0.043
	*p*-value	0.191	0.968	0.279	0.993	0.246	0.442	0.015	0.5	0.806
**Muscle Force Parameters (Women)**										
**Gait Parameters**		**Plantarflex**	**Dorsiflex**	**Kneeflex**	**Kneeex**	**Hipflex**	**Hipext**	**Hipabd**	**Hipadd**	**Handgrip**
Gait speed	Pearsons r	**0.486**	0.233	**0.524**	**0.443**	**0.502**	**0.527**	**0.432**	**0.538**	**0.419**
	*p*-value	0.006	0.207	0.002	0.013	0.004	0.002	0.015	0.002	0.019
Stride time	Pearsons r	−0.24	0.038	−0.298	−0.071	−0.283	−0.299	−0.209	−0.215	−0.146
	*p*-value	0.172	0.832	0.086	0.692	0.105	0.086	0.236	0.222	0.41
Stance time	Pearsons r	−0.3	0.094	**−0.350**	−0.142	**−0.361**	**−0.364**	−0.283	−0.322	−0.169
	*p*-value	0.085	0.595	0.042	0.424	0.036	0.034	0.104	0.064	0.338
Stride length	Pearsons r	0.309	0.037	0.254	**0.467**	0.312	**0.343**	0.227	0.326	0.152
	*p*-value	0.075	0.835	0.148	0.005	0.073	0.047	0.197	0.06	0.39
Step width	Pearsons r	**−0.489**	−0.014	**−0.416**	−0.234	**−0.415**	−0.303	**−0.543**	**−0.549**	**−0.369**
	*p*-value	0.003	0.938	0.014	0.182	0.015	0.082	0.001	0.001	0.032

Significant correlations (*p* < 0.05, *p* < 0.01) are marked in bold.

**Table 4:** Regression analysis between independent muscle group strength (predictors) and gait parameters (dependent variable). “x” marks included muscle groups.

Men					
	Gait Speed	Stride Time	Stance Time	Stride Length	Step Width
Plantarflexion	x		x		
Dorsiflexion					
Knee flexion		x	x		
Knee extension	x	x	x		
Hip flexion		x	x		
Hip extension	x	x	x		
Hip abduction		x			x
Hip adduction					
Handgrip				x	
R^2^	**0.273**	**0.443**	**0.413**	**0.195**	**0.166**
***p*** **-value**	**0.024**	**0.003**	**0.002**	**0.008**	**0.015**
**Women**					
	**Gait Speed**	**Stride Time**	**Stance Time**	**Stride Length**	**Step Width**
Plantarflexion	x				x
Dorsiflexion					
Knee flexion	x		x		x
Knee extension	x			x	x
Hip flexion	x		x		
Hip extension	x		x	x	x
Hip abduction	x				x
Hip adduction	x				
Handgrip	x				x
R^2^	0.352		0.151	**0.218**	**0.361**
*p*-value	0.215		0.172	**0.022**	**0.044**

Significant correlations (*p* < 0.05, *p* < 0.01) are marked in bold.

## Data Availability

The data presented in this study are available upon request from the corresponding author. The data are not publicly available to protect the participants’ confidentiality.

## References

[1] Ostrosky K.M., VanSwearingen J.M., Burdett R.G., Gee Z. (1994). A Comparison of Gait Characteristics in Young and Old Subjects. Phys. Ther..

[2] Shimada H., Kim H., Yoshida H., Suzukawa M., Makizako H., Yoshida Y., Saito K., Suzuki T. (2010). Relationship between Age-Associated Changes of Gait and Falls and Life-Space in Elderly People. J. Phys. Ther. Sci..

[3] JudgeRoy J.O., Davis I.B., Õunpuu S. (1996). Step Length Reductions in Advanced Age: The Role of Ankle and Hip Kinetics. J. Gerontol. A Biol. Sci. Med. Sci..

[4] Aboutorabi A., Arazpour M., Bahramizadeh M., Hutchins S.W., Fadayevatan R. (2016). The effect of aging on gait parameters in able-bodied older subjects: A literature review. Aging Clin. Exp. Res..

[5] Schwenk M., Howe C., Saleh A., Mohler J., Grewal G., Armstrong D., Najafi B. (2014). Frailty and Technology: A Systematic Review of Gait Analysis in Those with Frailty. Gerontology.

[6] Moreland J.D., Richardson J.A., Goldsmith C.H., Clase C.M. (2004). Muscle Weakness and Falls in Older Adults: A Systematic Review and Meta-Analysis. J. Am. Geriatr. Soc..

[7] AhmadiAhangar A., Javadian Y., Babaei M., Heidari B., Hosseini S., Aminzadeh M. (2018). The role of quadriceps muscle strength in the development of falls in the elderly people, a cross-sectional study. Chiropr. Man. Ther..

[8] Mignardot J.-B., Deschamps T., Barrey E., Auvinet B., Berrut G., Cornu C., Constans T., De Decker L. (2014). Gait disturbances as specific predictive markers of the first fall onset in elderly people: A two-year prospective observational study. Front. Aging Neurosci..

[9] Hamacher D., Liebl D., Hödl C., Heßler V., Kniewasser C.K., Thönnessen T., Zech A. (2018). Gait Stability and Its Influencing Factors in Older Adults. Front. Physiol..

[10] Pirker W., Katzenschlager R. (2017). Gait disorders in adults and the elderly: A clinical guide. Wien. Klin. Wochenschr..

[11] Buckinx F., Croisier J.L., Reginster J., Petermans J., Goffart E., Bruyère O. (2015). Relationship between Isometric Strength of Six Lower Limb Muscle Groups and Motor Skills among Nursing Home Residents. J. Frailty Aging.

[12] McGregor R.A., Cameron-Smith D., Poppitt S.D. (2014). It is not just muscle mass: A review of muscle quality, composition and metabolism during ageing as determinants of muscle function and mobility in later life. Longev. Healthspan.

[13] Fragala M.S., Alley D.E., Shardell M.D., Harris T.B., McLean R.R., Kiel D.P., Cawthon P.M., Dam T.-T.L., Ferrucci L., Guralnik J.M. (2016). Comparison of Handgrip and Leg Extension Strength in Predicting Slow Gait Speed in Older Adults. J. Am. Geriatr. Soc..

[14] Jabbar K.A., Seah W.-T., Lau L.K., Pang B.W.-J., Ng D.H.-M., Tan Q.L.-L., Chen K.K., Ullal J.M., Ng T.-P., Wee S.-L. (2021). Fast gait spatiotemporal parameters in adults and association with muscle strength—The Yishun study. Gait Posture.

[15] Burnfield J.M., Josephson K.R., Powers C.M., Rubenstein L.Z. (2000). The influence of lower extremity joint torque on gait characteristics in elderly men. Arch. Phys. Med. Rehabil..

[16] Inoue W., Ikezoe T., Tsuboyama T., Sato I., Malinowska K.B., Kawaguchi T., Tabara Y., Nakayama T., Matsuda F., Ichihashi N. (2017). Are there different factors affecting walking speed and gait cycle variability between men and women in community-dwelling older adults?. Aging Clin. Exp. Res..

[17] Bohannon R.W., Andrews A.W., Thomas M.W. (1996). Walking Speed: Reference Values and Correlates for Older Adults. J. Orthop. Sport. Phys. Ther..

[18] Herrero-Larrea A., Miñarro A., Narvaiza L., Gálvez-Barrón C., León N.G., Valldosera E., Felipe E., Valverde R.A., Kruse L., Sabater J.B. (2018). Normal limits of home measured spatial gait parameters of the elderly population and their association with health variables. Sci. Rep..

[19] Muehlbauer T., Granacher U., Borde R., Hortobágyi T. (2018). Non-Discriminant Relationships between Leg Muscle Strength, Mass and Gait Performance in Healthy Young and Old Adults. Gerontology.

[20] Guadagnin E.C., Priario L.A., Carpes F.P., Vaz M.A. (2019). Correlation between lower limb isometric strength and muscle structure with normal and challenged gait performance in older adults. Gait Posture.

[21] Demura T., Demura S.-I., Uchiyama M., Sugiura H. (2014). Examination of factors affecting gait properties in healthy older adults: Focusing on knee extension strength, visual acuity, and knee joint pain. J. Geriatr. Phys. Ther..

[22] Hollman J.H., McDade E.M., Petersen R.C. (2011). Normative spatiotemporal gait parameters in older adults. Gait Posture.

[23] Beauchet O., Allali G., Sekhon H., Verghese J., Guilain S., Steinmetz J.-P., Kressig R.W., Barden J.M., Szturm T., Launay C.P. (2017). Guidelines for Assessment of Gait and Reference Values for Spatiotemporal Gait Parameters in Older Adults: The Biomathics and Canadian Gait Consortiums Initiative. Front. Hum. Neurosci..

[24] Rowe E., Beauchamp M.K., Wilson J.A. (2021). Age and sex differences in normative gait patterns. Gait Posture.

[25] Frimenko R., Goodyear C., Bruening D. (2015). Interactions of sex and aging on spatiotemporal metrics in non-pathological gait: A descriptive meta-analysis. Physiotherapy.

[26] Bruening D.A., Baird A.R., Weaver K.J., Rasmussen A.T. (2020). Whole body kinematic sex differences persist across non-dimensional gait speeds. PLoS ONE.

[27] Bruening D.A., Frimenko R.E., Goodyear C.D., Bowden D.R., Fullenkamp A.M. (2015). Sex differences in whole body gait kinematics at preferred speeds. Gait Posture.

[28] Lewis C.L., Laudicina N.M., Khuu A., Loverro K.L. (2017). The Human Pelvis: Variation in Structure and Function During Gait. Anat. Rec..

[29] Ling C.H., de Craen A.J., Slagboom P.E., Gunn D.A., Stokkel M.P., Westendorp R.G., Maier A.B. (2011). Accuracy of direct segmental multi-frequency bioimpedance analysis in the assessment of total body and segmental body composition in middle-aged adult population. Clin. Nutr..

[30] Syed-Abdul M.M., Soni D.S., Barnes J.T., Wagganer J.D. (2021). Comparative analysis of BIA, IBC and DXA for determining body fat in American Football players. J. Sport. Med. Phys. Fit..

[31] Leardini A., Sawacha Z., Paolini G., Ingrosso S., Nativo R., Benedetti M.G. (2007). A new anatomically based protocol for gait analysis in children. Gait Posture.

[32] Trampisch U.S., Franke J., Jedamzik N., Hinrichs T., Platen P. (2012). Optimal jamar dynamometer handle position to assess maximal isometric hand grip strength in epidemiological studies. J. Hand Surg. Am..

[33] Schober P., Boer C., Schwarte L.A. (2018). Correlation Coefficients: Appropriate Use and Interpretation. Anesth. Analg..

[34] Lee H.-J., Chang W.H., Choi B.-O., Ryu G.-H., Kim Y.-H. (2017). Age-related differences in muscle co-activation during locomotion and their relationship with gait speed: A pilot study. BMC Geriatr..

[35] Hortobágyi T., DeVita P. (2000). Muscle pre- and coactivity during downward stepping are associated with leg stiffness in aging. J. Electromyogr. Kinesiol..

[36] Baudendistel S.T., Schmitt A.C., Stone A.E., Raffegeau T.E., Roper J.A., Hass C.J. (2021). Faster or longer steps: Maintaining fast walking in older adults at risk for mobility disability. Gait Posture.

[37] Kubinski S.N., McQueen C.A., Sittloh K.A., Dean J.C. (2015). Walking with wider steps increases stance phase gluteus medius activity. Gait Posture.

[38] van der Krogt M.M., Delp S.L., Schwartz M.H. (2012). How robust is human gait to muscle weakness?. Gait Posture.

[39] Goldberg E.J., Neptune R.R. (2007). Compensatory strategies during normal walking in response to muscle weakness and increased hip joint stiffness. Gait Posture.

[40] Whitcome K.K., Miller E.E., Burns J.L. (2017). Pelvic Rotation Effect on Human Stride Length: Releasing the Constraint of Obstetric Selection. Anat. Rec..

[41] Bailey C.A., Corona F., Pilloni G., Porta M., Fastame M.C., Hitchcott P.K., Penna M.P., Pau M., Côté J.N. (2018). Sex-dependent and sex-independent muscle activation patterns in adult gait as a function of age. Exp. Gerontol..

[42] Bruening D.A., Goodyear C.D., Bowden D.R., Barone R.E. (2015). Gender recognition from biologically guided anthropometric features. IJBM.

[43] Fessler D.M.T., Haley K.J., Lal R.D. (2005). Sexual dimorphism in foot length proportionate to stature. Ann. Hum. Biol..

[44] Cadore E.L., Rodríguez-Mañas L., Sinclair A., Izquierdo M. (2013). Effects of Different Exercise Interventions on Risk of Falls, Gait Ability, and Balance in Physically Frail Older Adults: A Systematic Review. Rejuvenation Res..

[45] Brown K.C., Hanson H.M., Firmani F., Liu D., McAllister M.M., Merali K., Puyat J.H., Ashe M.C. (2015). Gait Speed and Variability for Usual Pace and Pedestrian Crossing Conditions in Older Adults Using the GAITRite Walkway. Gerontol. Geriatr. Med..

[46] Malatesta D., Canepa M., Fernandez A.M. (2017). The effect of treadmill and overground walking on preferred walking speed and gait kinematics in healthy, physically active older adults. Eur. J. Appl. Physiol..

[47] Schmitt A.C., Baudendistel S.T., Lipat A.L., White T.A., Raffegeau T.E., Hass C.J. (2021). Walking indoors, outdoors, and on a treadmill: Gait differences in healthy young and older adults. Gait Posture.

